# Influence of Juiciness on *In Vivo* Aroma Release and Perception of Plant-Based Meat Analogue and Beef Patties

**DOI:** 10.1021/acs.jafc.4c13115

**Published:** 2025-04-03

**Authors:** Rutger Brouwer, Yifan Zhang, Elke Scholten, Ciarán G. Forde, Markus Stieger

**Affiliations:** † Food Quality and Design, Wageningen University & Research, 6700 AA Wageningen, The Netherlands; ‡ Physics and Physical Chemistry of Foods, Wageningen University & Research, 6700 AA Wageningen, The Netherlands; § Sensory Science and Eating Behaviour, Wageningen University & Research, 6700 AA Wageningen, The Netherlands

**Keywords:** meat analogues, meat alternatives, *in vivo* aroma release, aroma perception, PTR-MS, texture, flavor

## Abstract

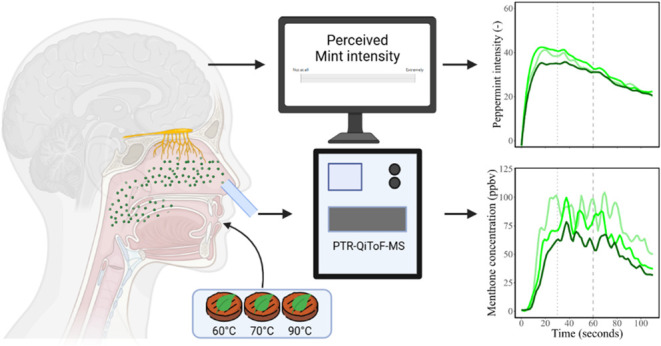

This study investigated how juiciness of the plant-based meat analogue (PBMA) and beef patties affects *in vivo* aroma release and perception. Patties were prepared from PBMA and beef mince, spiked with menthone as an aroma marker, and cooked to different core temperatures. Increasing core temperature decreased the perceived juiciness of PBMA and beef patties. *In vivo* in-nose menthone release and peppermint aroma perception were measured simultaneously using proton transfer reaction mass spectrometry and time–intensity profiling. Although differences in perceived juiciness were observed, no significant differences in *in vivo* aroma release and perception were observed in PBMA and beef patties, suggesting that aroma release and perception were not influenced by juiciness. Juiciness may not affect *in vivo* aroma release and perception due to the limited amount of serum released from the matrix during mastication and the entrapment of fat in the bolus. The effect of juiciness on aroma release may be product-specific.

## 1 Introduction

The excessive production and consumption of animal-based foods contribute to climate change. Climate change could be slowed down by reducing our reliance on animal-based foods and reverting to more plant-based diets.^1−3^ Plant-based meat analogues (PBMAs) have the potential to assist consumers in diversifying their dietary protein sources and reducing their intake of animal proteins. However, the palatability of currently available PBMAs is not comparable to meat products, with challenges associated with the juiciness, texture, and flavor release profile of many PBMAs. Several studies have already focused on aroma formation mechanisms during meat preparation and the interactions between the texture and flavor of meat.^4−9^ In contrast to meat, less is known about flavor formation in PBMAs. To the best of our knowledge, *in vivo* aroma release and perception of PBMAs have not been reported, and interactions between the texture and flavor of PBMAs are underexplored. A better understanding of the mechanisms underlying aroma release and perception during the consumption of plant-based meat analogues might contribute to the development of PBMAs with improved flavor properties.

Meat analogue patties are typically made from textured vegetable protein (TVP), nontextured protein, fat, binding agents (methylcellulose), flavorings, coloring agents, and water.^10^ During consumption, aroma compounds are released from the food matrix and reach the olfactory epithelial receptors via the retro-nasal pathway,^11^ resulting in the perception of aromas. Aroma partition coefficients and subsequent *in vivo* aroma release and perception are strongly influenced by food composition, texture, and matrix structure breakdown during consumption.^12−14^ The effect of the food matrix on *in vivo* aroma release and perception during consumption can be determined by combining temporal sensory methods (e.g., time–intensity profiling (TI)) with instrumental *in vivo* real-time aroma analysis methods (e.g., in-nose proton transfer reaction mass spectrometry (PTR-MS)).^15^ This provides the opportunity to quantitate the concentration of aroma compounds in the nasal cavity during food consumption and to relate the release of aroma compounds to their perception.^16−20^

Kaczmarska et al. already showed that aroma release between meat and meat analogues differs.^21^ When the headspace composition was compared, meat analogues had a more complex aroma composition and contained higher amounts of acids, alcohols, furans, and ketones than meat products. The presence of these compounds in PBMAs has been linked to the oxidation of unsaturated fatty acids, which often provides off-tastes and off-flavors in PBMAs, together with phenolic compounds, saponins, alkaloids, amino acids, and peptides.^22^ Flavor perception of PBMAs is not only driven by the volatile aroma composition but also by the release of the nonvolatile aroma compounds during consumption, which depends on the food matrix and textural properties. When PBMA and beef patties are masticated, liquid serum is released from the solid patty matrix into the oral cavity. The serum is released from the patty matrix at the early stages of mastication and contributes to perceived juiciness,^23,24^ which has been defined as “*the impression of moisture that a consumer experiences when chewing foods.*”^8,25^ Such serum release during consumption has been positively correlated to perceived juiciness.^23,24^ Serum composition may be relevant, as the relative amount of water and oil will determine which hydrophilic and hydrophobic taste and aroma compounds will be released.^23^ Perception is therefore a result of the interplay between texture, juiciness, and flavor release. For example, in the case of striploin steaks, a higher marbling increased juiciness, leading to increased release of serum during consumption and higher in-mouth volatile concentrations.^6,26^ In sausages, such an enhanced serum release and juiciness led to an increase in saltiness.^27^ Also, sweetness has been shown to increase by an increase in serum release, which was altered by changing the microstructure of food gels.^28^ However, such a relationship between juiciness, aroma release, and perception for PBMA and beef patties is currently not well understood.

The aim of this study was to determine how juiciness of plant-based meat analogue (PBMA) and beef patties affects *in vivo* aroma release and perception. We hypothesize that an increase in juiciness of PBMA and beef patties increases *in vivo* aroma release and perception since serum release might facilitate the release of aroma compounds. Juiciness of PBMA and minced beef patties was varied by changing the core cooking temperature during sous vide cooking. Juiciness intensity was quantified using Rank-Rating sensory tests. Descriptive texture and flavor profiles were obtained using the Rate-All-That-Apply (RATA) methodology. *In vivo* aroma release and perception were determined by PTR-MS using the in-nose menthone concentration as a marker of *in vivo* aroma release. Simultaneously, peppermint aroma intensity was evaluated during consumption using time–intensity (TI) profiling.

## 2 Materials and Methods

### 2.1 Sample Preparation

Beef and PBMA patties were prepared from commercially available minced beef (AH organic ground beef, Albert Heijn BV, The Netherlands) and minced PBMA (Beyond Meat Mince, The New Plant, The Netherlands). Beef patties were prepared by adding 4.55% (w/w) liquid whole egg (Vloeibaar Heelei Diepvries, Coco Vite, Belgium) and 0.45% (w/w) sodium chloride (NaCl, Salt Extra Fine, Jozo, The Netherlands) to the minced beef. The minced beef dough was hand-mixed in a stainless steel bowl for 60 s and then removed from the bowl and thoroughly mixed by hand for 120 s. The beef dough was shaped manually into balls of approximately 12 g, which has previously been determined to be the bite size of beef and PBMA patties.^23^ The minced PBMA dough was kneaded with a spatula in a stainless steel bowl for 90 s. The stainless steel bowl was placed in an ice bath to prevent the melting of saturated fats. After being mixed, the PBMA dough was shaped manually into balls of approximately 12 g. Menthone (154.25 g mol^–1^, log *P* = 2.7, L-Menthone, Sigma-Aldrich, Merck KGaA, Germany) was added (0.02% w/w) to the minced balls with a pipette by injecting the liquid menthone into the center of the meatballs. Menthone was chosen as an aroma marker since it provides a distinct and recognizable peppermint aroma that is otherwise absent in the patties, allowing to track the aroma perception (peppermint intensity) that is associated with the release of the specific aroma compound (menthone) during consumption. The selection of menthone as the traceable aroma compound was based on its instrumental detectability, the absence of menthone mass in the headspace of PBMA and beef patties, and the distinct aroma quality of peppermint during consumption. The balls were then shaped manually into small, bite-sized patties (36 mm diameter; 20 mm height; 12 g) to allow the entire patty to be consumed within one bite. This procedure ensured that the amount of menthone was equal across patties. Menthone was only added to the patties used for the PTR-MS and sensory time–intensity (TI) evaluations. Patties without menthone were evaluated in the Rank-Rating (juiciness) and RATA sensory evaluations. The raw PBMA and beef patties spiked with menthone were packed in vacuum bags (Black & Orange, Disposable Discounter, The Netherlands), from which 95% of air was removed and then sealed (Henkovac M2, The Netherlands). The patties were allowed to rest at 4 °C for 24 h. The small, bite-sized patties were cooked sous vide to different core temperatures. Vacuum-packed patties were placed into a preheated water bath at temperatures of 60, 70, or 90 °C for 60 min. Patties were subsequently removed from the plastic bags, transferred into foam boxes, and cooled down to a core temperature of 60 °C (10 min cooling time for patties cooked at 90 °C, 5 min for patties cooked at 70 °C, and 0 min for patties cooked at 60 °C). After cooling, the patties were grilled on a double-plate grill (DeLonghi, Italy) at 200 °C for 25 s, with a distance of 2 cm between the two heating plates. After grilling, the PBMA patties were allowed to cool in the foam box for 1 min, and the beef patties for 4 min, to reach a serving temperature of 55 °C before being served to participants or instrumentally characterized. The menthone headspace concentration was quantified in PBMA and beef patties after sous vide cooking using PTR-MS. PBMA and beef patties were spiked with five concentrations of menthone (0, 0.01, 0.1, 0.5, 1%) and cooked sous vide for 60 min at 60, 70, and 90 °C. Patties were placed into glass vials after cooking, incubated at 37 °C in an autosampler, and the headspace concentration of menthone was determined (PTR-MS). The headspace concentration of menthone did not significantly differ between patties cooked to different core temperatures (data not shown), ensuring that the menthone concentrations of all patties consumed in the *in vivo* aroma release and perception study were similar. The patties are coded according to their core cooking temperature as PBMA60, PBMA70, PBMA90, BEEF60, BEEF70, and BEEF90. Sample names refer to the core temperature of the patties, and all patties were served at 55 °C. All ingredients used were food-grade, and samples were prepared and served in a food-safe environment and followed a safe-for-consumption protocol.

### 2.2 Participants

The data collection involved two sensory panels. The first panel was used for Rank-Rating and RATA sensory evaluations. This panel consisted of *n* = 59 naïve participants (38 females) aged 26 ± 4 years. Participants were recruited via posters, word-of-mouth, and online recruitment. Participants were screened to have no dental or swallowing issues, were nonsmokers, had no reported taste or smell issues and no allergies, had experience with using a computer, and had good general health, were not vegetarian or vegan, and were willing to eat meat and plant-based meat, all based on self-reported data.

The second panel was used for the quantitation of *in vivo* aroma release and perception using PTR-MS and TI-profiling and consisted of 12 females (25 ± 3 years old) who did not participate in the first panel. This panel was kept as homogeneous as possible to reduce interindividual variation in mastication behavior and to limit interindividual differences in aroma release.^29^ Participants had to comply with the following inclusion criteria (self-reported): no dental issues, no swallowing issues, no smoking habits, no taste or smell issues, no allergies, experience with using a computer, good general health, female, Caucasian, BMI between 18.5 and 20 kg/m^2^, between 18 and 30 years old, and not vegetarian or vegan. Only females were included to reduce heterogeneity and to increase sensory sensitivity.^30,31^ After complying with the inclusion criteria, 18 participants joined a screening session, and 12/18 screened participants were selected based on their stimulated salivary flow rate (1.2 ± 0.4 g/min), natural consumption time of PBMA and beef patties (12 g) (25 ± 6 s), and the liking of the patties used in this study to reduce interindividual variation. Participants were included when their salivary flow rate and natural consumption time of PBMA and beef patties were within two SDs of the mean of the panel. Participants attended an information session in which the tasks of the study were explained without revealing the goal of the study. During the information session, participants had the opportunity to ask questions about the study. All participants signed an informed consent form, were free to withdraw from the study at any time, and received financial compensation for their participation after completion. The study did not meet the requirements to be reviewed by the Medical Research Ethical Committee of The Netherlands according to the “Medical Research Involving Human Subjects Act” of The Netherlands (WMO in Dutch). The study was conducted in agreement with the ethics regulations laid out in the Declaration of Helsinki (2013).

### 2.3 Rank Rating of Juiciness

The rank–ranking procedure was explained and executed in three separate sessions. First, each participant attended a 60 min familiarization session, in which they were introduced to the study and got familiarized with the Rank-Rating procedure. Afterward, participants joined two sessions of 60 min each, in which they evaluated beef patties (BEEF60, BEEF70, BEEF90) or PBMA patties (PBMA60, PBMA70, PBMA90). The juiciness of the PBMA and beef patties was quantified separately using Rank-Rating tests. Half of the participants started with a beef patty session, while the other half started with PBMA patties, and this selection was randomized. Three patties (12 g, serving temperature 55 °C) were presented simultaneously on a preheated plate (70 °C) to the participants (*n* = 59). Patties were coded in a randomized order with 3-digit codes. Participants were instructed to taste the patties and evaluate the juiciness intensity. Juiciness was defined as the sensation of moisture, juice, or liquid being released from food during consumption. Participants were asked to rank the juiciness intensity by placing the samples on a 100 mm unstructured line scale, which was anchored from ″not juicy″ to ″very juicy.″ Participants were not required to cleanse their palates between the three patties but were instructed to cleanse their palates with water and crackers after the Rank-Rating procedure was completed. The Rank-Rating data was collected using EyeQuestion software (EyeQuestion software, version 5.11.2, Logic8, Netherlands).

### 2.4 Rate-All-That-Apply (RATA)

Participants performed a RATA evaluation to obtain descriptive sensory profiles for all patties. Texture, taste, and flavor perception were assessed by *n* = 59 participants who previously performed the Rank-Rating evaluation. The RATA was explained and executed in three separate sessions. First, each participant attended a 60 min familiarization session, in which they were introduced to the study, got familiarized with the RATA procedure, and received a list of sensory attributes, including definitions. All attributes were explained with a special emphasis on juiciness, and finally, the participants received PBMA70 and BEEF70 to familiarize themselves with the samples and the attribute list. The definitions of the 13 texture, taste, and flavor attributes are provided in Table 1. The samples were presented to participants in a sequential monadic presentation in random order in disposable cups labeled with 3-digit codes. Participants were instructed to eat the patties (12 g, serving temperature 55 °C) and select the attributes that apply to describe the perception of each sample. The 13 attributes were evaluated in two blocks, starting with a block of texture attributes, followed by a block of taste and flavor attributes. The order of attributes within each block was randomized over participants but was kept constant across samples per participant per session. Texture attributes were evaluated after the first bite. After further chewing, participants evaluated the taste and flavor attributes. After selecting an attribute, the intensity of the selected attribute was assessed on a 9-point scale anchored from “low” to “high” intensity. Participants were instructed to cleanse their palates with water and crackers between samples. The RATA data was collected using EyeQuestion software (EyeQuestion software, version 5.11.2, Logic8, The Netherlands).

**Table 1:** Sensory Attributes and Definitions Used for RATA Evaluations

Attribute	Definition
*Texture*	
Juiciness	Sensation of moisture/juice/liquid being released from food during consumption.
Dryness	Sensation of dryness in mouth.
Hardness	Force applied by the (molar) teeth to bite through the food.
Chewiness	Effort required to masticate the food until it is ready to be swallowed.
Tenderness	Sensation related to how easily the food is chewed or cut and how soft it is.
Crumbliness	Extent to which the food breaks up into particles in the mouth during the first few chews.
Fibrousness	Sensation of elongated structures in the food associated with the presence of fibers.
Fattiness	Sensation of fat in the mouth.
*Taste*	
Saltiness	Salty taste sensation.
Umami	Savory, broth-like taste sensation.
*Flavor*	
Meat flavor	Flavor of meat, related to products like beef, chicken, or pork.
Beany flavor	Flavor related to beans and legumes.
Off-flavor	General sensation of unpleasant aromas or tastes.

### 2.5 *In Vivo* Nose-Space PTR-MS-Analysis

The *in vivo* nose-space aroma release and TI data collection were explained and executed in six sessions over a period of one month. First, participants were screened and trained in three sessions of 60 min. After selecting eligible participants (*n* = 12), *in vivo* aroma release and dynamic aroma perception were quantified during three sessions of 90 min. The screening session included an explanation of the study, a familiarization with the patties, and multiple tests to screen the panel. Details of this screening can be found in Section 2.2. During the screening session, participants consumed PBMA60, PBMA90, BEEF60, and BEEF90 to be familiarized with the samples and the differences in the sample set. All samples were coded with random 3-digit codes. During the two training sessions, participants were acquainted with the consumption protocol, the *in vivo* aroma release procedure, and the TI methodology. The first training session introduced participants to the consumption protocol. Moreover, the participants practiced the consumption protocol with all samples to ensure that the mastication protocol was appropriate for each sample set. The pace of the chewing protocol was based on previous studies,^20^ while the length of the mastication process was based on the natural consumption time of the participants. Participants were instructed not to eat, drink (except for water), or brush their teeth in the 2 h preceding their sessions. Participants were asked not to wear perfume or a strong-smelling lotion. At the start of each session, participants received a blank sample with a core temperature of 70 °C as a warm-up sample. The *in vivo* nose-space aroma release was measured using a high-sensitivity PTR-QiToF-MS (Ionicon Analytik, Innsbruck, Austria). The device operated at drift tube temperatures, voltages, and pressures of 100 °C, 900 V, and 460 Pa, respectively, resulting in a field density ratio (E/N) of 133 Td. The volatile compounds present in the nose space were introduced into the system through a PEEK capillary line heated to 100 °C with a flow rate of 40 mL/min. The PTR-MS was used in SCAN mode over a mass range of *m*/*z* 0–500 with an acquisition rate of 1 s. Laboratory air was measured for at least 20 s before every measurement. Participants were instructed to connect to the PTR-MS by inserting two Teflon tubes (diameter: 6.8 mm; length: 6.4 cm, connected to the heated inlet tubes) into their nostrils and to breathe normally through their nose. Their breath was first sampled for at least 30 s, and participants received a patty (12 g). Samples were transported from the grill to the participants in a foam box to control the serving temperature. The participants used a cocktail stick to place the entire bite-sized patties (12 g) into their mouths. The sample was chewed for 35 s for beef and 30 s for PBMA patties at a chewing frequency of 1 chew/s. The total chewing time was different for beef and PBMA because the texture of the patties was different and especially BEEF90 required more chews. This study does not compare beef and PBMA directly; therefore, the different chewing regimes do not influence the outcome of the study. A metronome guided the chewing frequency of the participants. At the end of the chewing period, participants swallowed the patty. One swallow was not sufficient to completely swallow the bolus, so participants were instructed to swallow at least two times. The second swallow occurred 30 s after the first swallow. Participants were instructed not to chew after the first swallow. If necessary, participants could freely swallow for a third time. When swallowing, participants always raised their hands. This allowed the researcher to record the times of the swallowing moments. After the second swallow, participants stayed connected to PTR-MS for another 90 s, and the aroma release and perception were still measured. The total sampling time for each sample was 180 s. Participants were instructed to use cold water, hot water, and a tongue scraper to cleanse their palates for at least 3 min between samples. Other palate cleansers were not suitable for this study because they could affect the volatile release of the following measurements. Participants (*n* = 12) evaluated every sample in triplicate. The replicates were assessed over the course of three sessions on different days. Samples were coded with three-digit codes and presented in a random order within each session.

### 2.6 Data Extraction and Peak Selection

Mass peaks at *m*/*z* 155.137 corresponding to menthone and its main fragments at *m*/*z* 137.117, 95.088, and 81.070 were extracted.^32,33^ The concentration (ppbV) of menthone and its main fragments was extracted with PTR-MS Viewer software (PTR-MS Viewer 3.4.2.1, Ionicon Analytik, Innsbruck, Austria). A release curve was constructed for the sum of *m*/*z* 155.137 and its main fragments by plotting the concentration (ppbV) versus time (s). Each release curve was divided into five segments: lab air (1–20 s), breath (20–50 s), consumption (50–85 s), between first and second swallow (85–115 s), and postswallow (second swallowing point until 180 s). Each part of the curve was averaged across participants and replicates to obtain an average release curve for each patty. Three parameters were extracted from each individual release curve: the total amount of released menthone as the area under the curve (AUC_R), the maximum released concentration of menthone (*I*_max__R), and the time to reach the maximum released concentration of menthone (*T*_max__R). After extraction of the individual values for AUC_R, *I*_max__R, and *T*_max__R, the corresponding averages were obtained across participants and replicates.

### 2.7 Time–Intensity Profiling

The peppermint aroma intensity was determined by using the TI methodology for beef and PBMA patties. The TI-profiling was conducted simultaneously with the *in vivo* nose-space analysis using the PTR-MS. Participants (*n* = 12; triplicate) were instructed to place the entire patty (12 g) in the mouth and simultaneously click the start button on a screen. Participants continuously scored the peppermint aroma intensity over time by moving the cursor horizontally on a 100 mm unstructured line scale anchored from “not at all” to “very” intense (EyeQuestion software, version 5.11.2). The total duration of the evaluation was set at 140 s, indicating that participants evaluated peppermint intensity during chewing (30–35 s for PBMA and beef, respectively), between swallow one and swallow two (30 s), and after the patty had been swallowed completely (approximately 75–80 s). Intensity scores were recorded at intervals of 0.5 s. Three parameters were extracted from the individual TI curves: the total sensory intensity of peppermint aroma as the area under the curve (AUC_S), the maximum sensory intensity of peppermint aroma (*I*_max__S), and the time to reach the maximum sensory peppermint aroma intensity (*T*_max__S). The present study used the standardization described by Liu and MacFie to correct for individual curves.^34^ After the extraction of the individual values for AUC_S, *I*_max__S, and *T*_max__S, the corresponding averages were obtained across participants and replicates.

### 2.8 Statistical Data Analyses

Rank-Rating and RATA data were reported as mean values with a standard deviation (SD). Significant differences between PBMA and beef patties cooked to different core temperatures were determined using linear mixed models (LMM) with the lme4 package^35^^35^ followed by Tukey posthoc analyses using the rstatix package.^36^ Separate LMMs were executed for the PBMA and beef patties, as the direct comparison of those products was not the focus of the study, and the sensory data of those patties was, on purpose, collected in separate sessions. The LLM treated samples (3) as fixed factors and participants (59 for Rank Rating and RATA) as random factors. Correlations between sensory attributes were assessed by Pearson correlation analysis using the PerformanceAnalytics package.^37^ Pearson correlation coefficients were determined separately for PBMA and beef patties.

PTR-MS and TI results were reported as mean values with a standard error (SE). Averaged release curves were plotted for *m*/*z* = 155.127 against consumption time for PBMA and beef separately using the ggplot2 package.^38^ The average perceived intensity of peppermint aroma was plotted against consumption time for PBMA and beef separately. All curves were plotted without SE to improve readability. LLMs were performed separately for PBMA and beef patties to investigate the effect of core temperature (juiciness) on the release and perception of menthone intensity. Core temperature was set as a fixed effect, and participants, replicates, and order were set as random effects. All statistical data analyses were performed at a significance level of *p* < 0.05 using *R* software.^39^

## 3 Results and Discussion

### 3.1 Rank Rating of Juiciness Intensity

**Figure 1:**
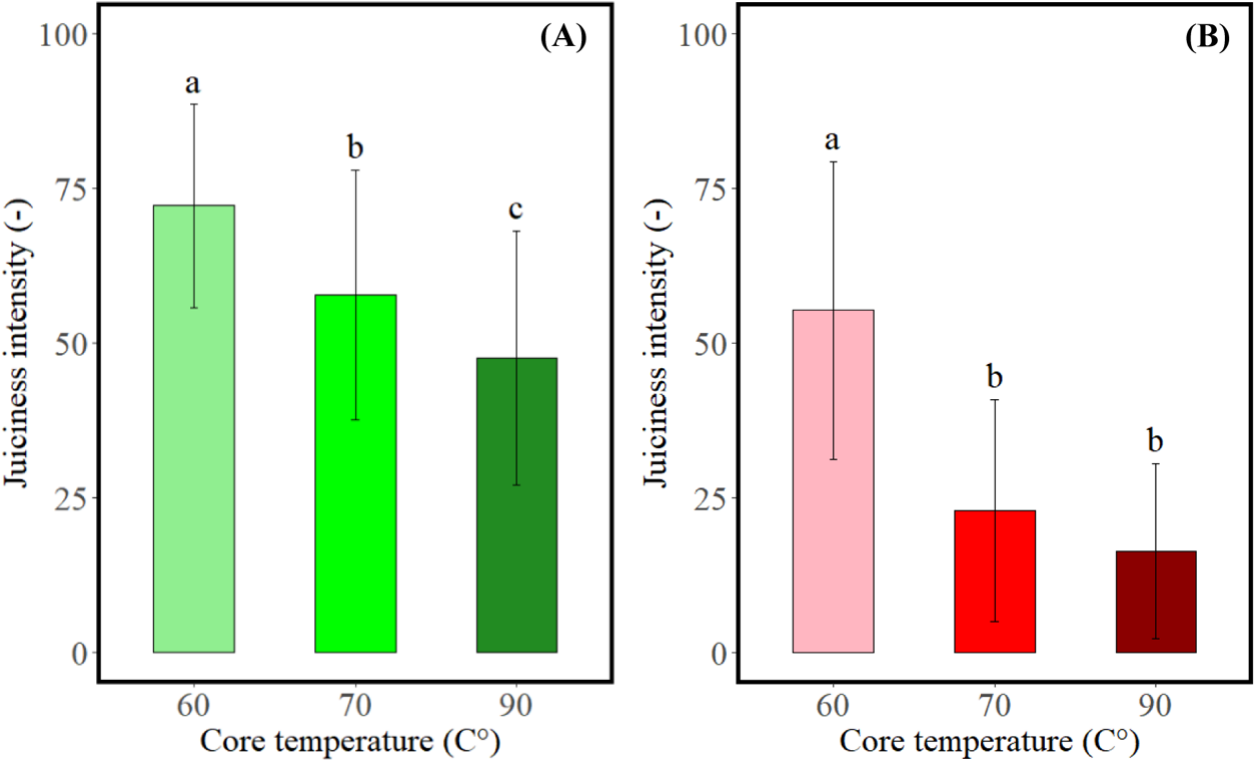
Mean juiciness intensity (±SD) of the Rank-Rating evaluation for (A) PBMA patties (*n* = 59) and (B) beef patties (*n* = 59) cooked to three core temperatures. Different letters indicate significant differences between means (*p* < 0.05).

The mean juiciness intensity ratings obtained by the Rank-Rating methodology are summarized in Figure 1. Increasing the core temperature significantly decreased the juiciness intensity for PBMA (*F* = 20.34, *p* < 0.001) and beef (*F* = 64.89, *p* < 0.001) patties. For PBMA patties, three significantly different levels of juiciness intensity were obtained, while for beef patties, two significantly different levels of juiciness were obtained.

Juiciness intensity of PBMA patties decreased by 20% when the core temperature increased from 60 to 70 °C and by 34% when the core temperature increased from 60 to 90 °C. For beef patties, juiciness intensity decreased by 59% when the core temperature increased from 60 to 70 °C and by 71% when the core temperature increased from 60 to 90 °C. The findings of the current study agree with previous studies reporting that with increasing core temperature, juiciness intensity decreases in PBMAs and meat. Increasing the core temperature from 60 to 90 °C leads to an increase of around 10% in cooking loss in similarly prepared patties, which is accompanied by a decline in juiciness.^23^ To summarize, PBMA and beef patties differing in juiciness intensity were obtained from the same starting materials (raw doughs) by varying the core cooking temperature during sous vide cooking, which allows us to examine the effect of juiciness on *in vivo* aroma release and perception.

### 3.2 Sensory Properties of Plant-Based Meat Analogue and Beef Patties

The intensities of texture, taste, and flavor attributes of PBMA patties are summarized in Table 2.

**Table 2:** Intensity Scores (Mean ± SD) of PBMA Patties Obtained from the RATA Evaluations for Texture, Taste, and Flavor Attributes (*n* = 59)^a^

	*PBMA60*	*PBMA70*	*PBMA90*	*F*	*p*
***Texture***					
Juiciness	7.0 ± 1.2 a	6.1 ± 1.6 b	5.3 ± 1.8 c	16.5	<0.001
Dryness	1.8 ± 2.2 b	2.4 ± 2.6 ab	2.9 ± 2.7 a	4.4	<0.05
Hardness	2.7 ± 2.1 ab	3.2 ± 2.3 a	2.3 ± 1.8 b	7.9	<0.001
Chewiness	4.6 ± 2.3 ab	5.0 ± 2.1 a	4.1 ± 2.0 b	4.8	<0.05
Tenderness	5.7 ± 2.0 a	4.9 ± 2.5 b	6.1 ± 1.8 a	6.9	<0.01
Crumbliness	4.4 ± 2.9	4.6 ± 2.8	4.6 ± 2.8	0.3	0.750
Fibrousness	4.0 ± 2.7 ab	4.5 ± 2.7 a	3.5 ± 2.7 b	5.9	<0.01
Fattiness	5.1 ± 2.5 a	4.8 ± 2.4 ab	4.2 ± 2.3 b	4.6	<0.05
***Taste***					
Saltiness	4.4 ± 2.1	4.2 ± 2.1	4.2 ± 2.1	0.9	0.393
Umami	5.8 ± 1.9	5.6 ± 2.0	5.8 ± 1.8	1.0	0.387
***Flavor***					
Meat flavor	4.6 ± 2.7	4.4 ± 2.6	4.2 ± 2.5	1.1	0.326
Beany flavor	3.6 ± 2.2	4.0 ± 2.5	4.0 ± 2.5	0.9	0.394
Off-flavor	0.7 ± 1.2 a	1.1 ± 1.7 ab	1.2 ± 1.6 b	5.3	<0.01

a Different lower-case letters indicate significant differences between patties for an attribute (*p* < 0.05). *F* and *p* values are derived from linear mixed models with samples as fixed factors and participants as random effects.

With increasing core temperature, the juiciness intensity of PBMA patties significantly decreased (*F* = 16.5, *p* < 0.001), confirming the Rank-Rating results (Figure 1). PBMA patties significantly differed in dryness (*F* = 4.4, *p* < 0.05), hardness (*F* = 7.9, *p* < 0.001), chewiness (*F* = 4.8, *p* < 0.05), tenderness (*F* = 6.9, *p* < 0.01), fibrousness (*F* = 5.9, *p* < 0.01), fattiness (*F* = 4.6, *p* < 0.05), and off-flavor (*F* = 5.3, *p* < 0.01). As expected, juiciness was negatively correlated to dryness (*r* = −0.45). Higher core temperatures probably led to higher cooking loss leading to drier patties. Juiciness was positively correlated to fattiness (*r* = 0.42) (Table 4A). The fact that fattiness and juiciness are correlated has been previously shown for similar patties.^23^ As juiciness is related to both fattiness and serum release, fattiness has also been shown to be positively correlated with serum release.^23^ While texture differences between PBMA patties varying in core temperature were significant for several attributes, the differences in intensity were small (typically <1.0 on a 9-point scale) and did not follow a consistent pattern as a function of core temperature. Juiciness therefore did not significantly correlate with any texture attribute except fattiness. The limited differences in texture perception between patties varying in core temperature and juiciness may be related to the TVP used in the preparation of the PBMA patties. As TVP was already denatured before incorporation into the PBMAs,^42^ cooking temperature did not affect the structure and the texture of the PBMA patty, and therefore differences in texture were also limited.

With respect to flavor attributes, juiciness has previously been shown to be positively correlated with saltiness (*r* = 0.24) and savoriness (*r* = 0.15) and negatively correlated with beany flavor (*r* = −0.20) in PBMA patties.^43^ In our study, decreasing juiciness significantly increased the intensity of plant-related off-flavors but showed no significant impact on umami, saltiness, meat, and beany flavor intensity. This may again be attributed to limited differences in texture, even though differences in juiciness were significant.

The intensities of texture, taste, and flavor attributes of beef patties are summarized in Table 3.

**Table 3:** Intensity Scores (Mean ± SD) of Beef Patties Obtained from the RATA Evaluations for Texture, Taste, and Flavor Attributes (*n* = 59)^a^

	*BEEF60*	*BEEF70*	*BEEF90*	*F*	*p*
***Texture***					
Juiciness	6.2 ± 1.6 a	3.3 ± 2.0 b	2.1 ± 1.8 c	93.7	<0.001
Dryness	2.5 ± 2.5 c	5.6 ± 2.8 b	6.7 ± 2.1 a	70.6	<0.001
Hardness	3.7 ± 2.2 c	5.5 ± 2.1 a	4.6 ± 2.7 b	13.2	<0.001
Chewiness	5.2 ± 1.9 b	6.2 ± 2.0 a	5.8 ± 1.8 a	5.6	<0.01
Tenderness	4.3 ± 2.2 a	3.0 ± 2.2 b	3.2 ± 2.3 b	8.9	<0.001
Crumbliness	3.8 ± 2.3 b	4.3 ± 2.7 b	4.7 ± 2.6 a	8.9	<0.001
Fibrousness	4.3 ± 2.9	4.6 ± 3.1	4.1 ± 3.0	0.4	0.670
Fattiness	5.9 ± 2.1 a	4.8 ± 2.4 b	3.8 ± 2.5 c	21.3	<0.001
***Taste***					
Saltiness	4.6 ± 1.9 a	4.4 ± 1.9 a	3.4 ± 2.3 b	9.4	<0.001
Umami	4.9 ± 2.6 a	4.2 ± 2.7 b	3.8 ± 2.7 b	10.1	<0.001
***Flavor***					
Meat flavor	7.9 ± 1.3 a	7.7 ± 1.5 ab	7.4 ± 1.4 b	4.4	<0.05
Beany flavor	0.5 ± 0.9 b	0.6 ± 1.0 ab	1.0 ± 1.8 a	4.8	<0.01
Off-flavor	0.8 ± 1.4	0.6 ± 1.1	0.8 ± 1.6	0.2	0.800

a Different lower-case letters indicate significant differences between patties for an attribute (*p* < 0.05). *F* and *p* values are derived from linear mixed models with samples as fixed factors and participants as random effects.

For beef patties, juiciness intensity also significantly decreased with increasing core temperature (*F* = 93.7, *p* < 0.001), confirming the results of the Rank-Rating evaluation. As explained previously for the PBMA patties, this can probably be attributed to a higher cooking loss at higher core temperatures, which also explains the significant differences in dryness between beef patties differing in core temperature. In addition, hardness, chewiness, tenderness, crumbliness, fattiness, saltiness, umami, meat flavor, and beany flavor significantly differed between beef patties differing in core temperature. In contrast to the PBMA patties, these attributes were correlated to the juiciness of beef patties. Next to the expected negative correlation between juiciness and dryness (*r* = −0.69), juiciness was positively correlated with tenderness (*r* = 0.44), fibrousness (*r* = 0.15), and fattiness (*r* = 0.49) and negatively correlated with hardness (*r* = −0.23) and chewiness (*r* = −0.19) (Table 4B). The correlations are in line with previous studies that reported the descriptive sensory profiles of meat cooked at different temperatures.^40,41,44−46^ Core cooking temperature had a stronger effect on the texture perception of beef patties compared with that of PBMA patties, most likely due to the difference in structure between beef and PBMA patties. When heated, myofibrillar meat proteins shrink and denature, which leads to large changes in the structure, which has been shown to provide a tougher meat texture.^47^ This explains why correlations for beef patties were more pronounced than for meat analogue patties, as TVPs do not considerably change their structure upon heating. Also, for taste and flavor attributes, significant differences were observed among beef patties, most likely also as a result of larger differences in structure. With decreasing core temperature (increasing juiciness) of beef patties, saltiness, umami, and meat flavor increased, whereas beany flavor intensity decreased. The correlation between juiciness and saltiness aligns with previous studies that reported enhanced taste and flavor perception for increased serum release.^6,26−28^ The correlation between juiciness and taste and aroma perception can most likely be attributed to differences in serum release during consumption. An increased serum release potentially enhanced the release of tastants and aroma compounds from the patty matrix into the oral cavity, facilitating the transport of tastants such as salt, umami, and aroma compounds to the taste buds, causing a more intense taste and flavor perception.

**Table 4:** Pearson Correlation Coefficients of Texture, Flavor, and Taste Attributes of the RATA Evaluation of PBMA (A) and Beef Patties (B)^a^

(A) PBMA	Juiciness	Dryness	Hardness	Chewiness	Tenderness	Crumbliness	Fibrousness	Fattiness	Saltiness	Umami	Meat flavor	Beany	Off-flavor
Juiciness	1	–0.45***	n.s.	n.s.	n.s.	n.s.	n.s.	0.42***	0.24**	n.s.	n.s.	–0.20*	n.s.
Dryness		1	0.35***	n.s.	n.s.	0.26**	0.21*	n.s.	n.s.	n.s.	0.28**	n.s.	0.18*
Hardness			1	0.31***	n.s.	0.31***	0.22**	n.s.	0.23**	n.s.	0.36***	n.s.	0.20*
Chewiness				1	n.s.	n.s.	0.25**	n.s.	n.s.	n.s.	n.s.	n.s.	n.s.
Tenderness					1	0.19*	n.s.	n.s.	0.18*	0.17*	n.s.	n.s.	n.s.
Crumbliness						1	n.s.	n.s.	n.s.	n.s.	n.s.	n.s.	n.s.
Fibrousness							1	n.s.	n.s.	n.s.	n.s.	0.20*	0.22**
Fattiness								1	0.33***	n.s.	0.29***	–0.21*	n.s.
Saltiness									1	n.s.	0.28***	n.s.	n.s.
Umami										1	n.s.	–0.23**	–0.19*
Meat flavor											1	–0.38***	n.s.
Beany												1	n.s.
Off-flavor													1

(B) Beef	Juiciness	Dryness	Hardness	Chewiness	Tenderness	Crumbliness	Fibrousness	Fattiness	Saltiness	Umami	Meat flavor	Beany	Off-flavor
Juiciness	1	–0.69***	–0.23**	–0.19*	0.44***	n.s.	n.s.	0.49***	0.35***	0.37***	0.23**	n.s.	n.s.
Dryness		1	0.39***	0.29***	–0.22**	n.s.	n.s.	–0.41***	n.s.	–0.24**	–0.17*	n.s.	0.23**
Hardness			1	0.33***	n.s.	n.s.	n.s.	n.s.	n.s.	n.s.	n.s.	n.s.	0.20*
Chewiness				1	–0.30***	0.17*	0.26**	n.s.	n.s.	n.s.	n.s.	n.s.	n.s.
Tenderness					1	0.17*	0.23**	0.31***	0.28***	0.30***	n.s.	n.s.	n.s.
Crumbliness						1	0.24**	0.17*	0.25**	0.34***	n.s.	0.21*	n.s.
Fibrousness							1	0.29***	0.29***	0.49***	n.s.	n.s.	n.s.
Fattiness								1	0.29***	0.36***	0.21**	n.s.	n.s.
Saltiness									1	0.44***	0.17*	0.19*	n.s.
Umami										1	0.29***	n.s.	n.s.
Meat flavor											1	–0.32***	–0.16*
Beany												1	0.30***
Off-flavor													1

a Significant correlations are depicted by an asterisk. (*) *p* < 0.05, (**) *p* < 0.01, (***) *p* < 0.001, and (n.s.) not significant.

### 3.3 In-Nose Menthone Release and Dynamic Peppermint Perception of Plant-Based Meat Analogue and Beef Patties

**Figure 2:**
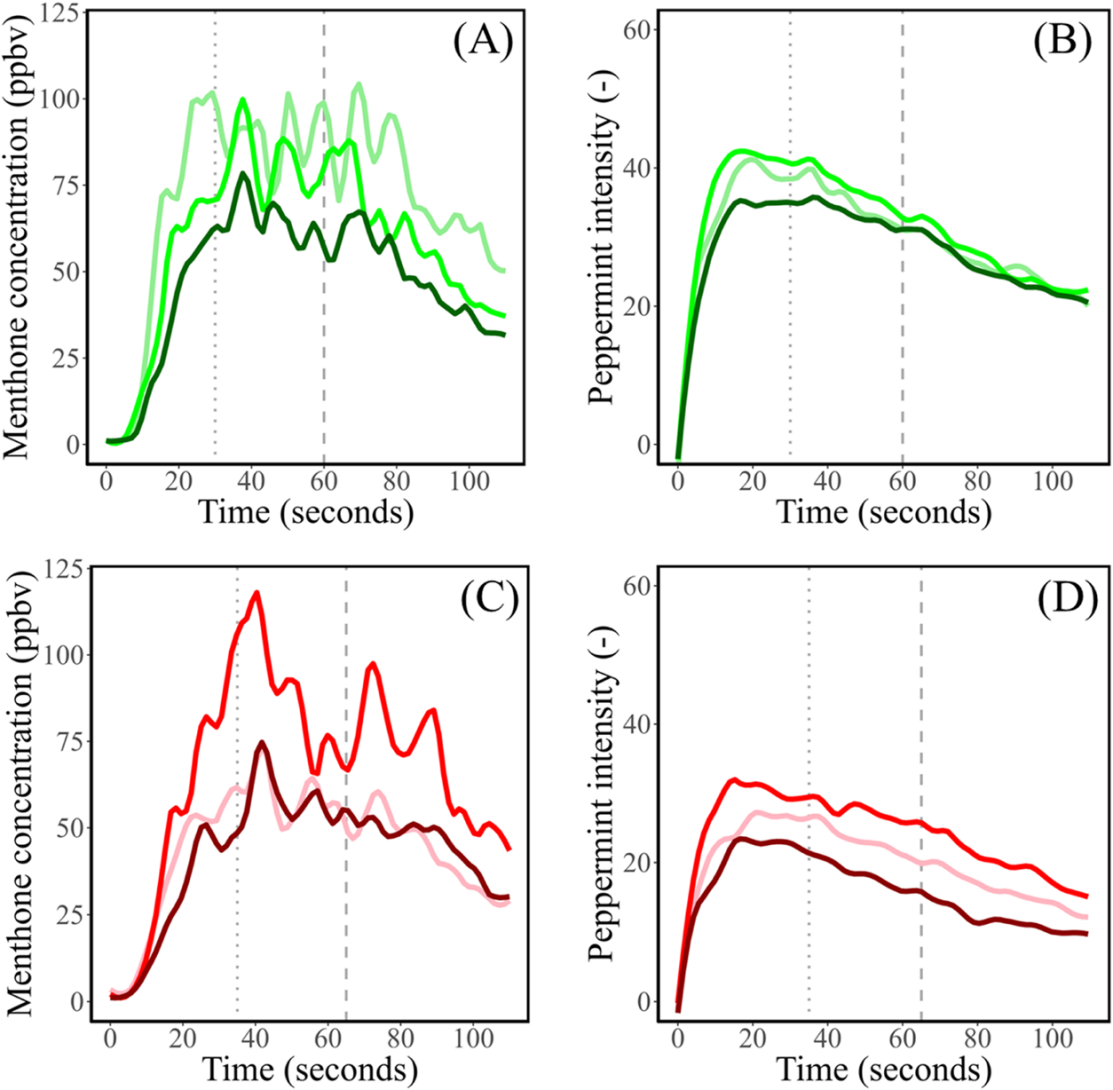
Average (*n* = 12, triplicate) *in vivo* release of menthone (*m*/*z* = 155.137 + fragments) (A) and perceived peppermint aroma intensity (B) during and after consumption of plant-based meat analogue patties differing in core temperature (60 °C: light green; 70 °C: green; 90 °C: dark green) and *in vivo* release of menthone (*m*/*z* = 155.137 + fragments) (C) and perceived peppermint aroma intensity (D) of beef patties differing in core temperature (60 °C: light red; 70 °C: red; 90 °C: dark red) of beef patties. The first and second moments of swallowing are depicted by the dotted and dashed lines, respectively.

The averaged *in vivo* menthone release curves and perceived peppermint intensity during consumption of PBMA and beef patties are depicted in Figure 2. Table 5 summarizes the parameters extracted from the dynamic release and perception curves for the PBMA and beef patties (mean ± SE).

**Table 5:** Parameters (Mean ± SE) Extracted from the *In Vivo* Menthone Release (PTR-MS) and Peppermint Aroma Intensity Perception (TI) Curves during Consumption (*n* = 12, Triplicate) of PBMA (A) and beef (B) Patties^a^

(A) PBMA	*PBMA60*	*PBMA70*	*PBMA90*	*F*	*p*
*I*_max_					
*I*_max__R (ppbV)	138 ± 24	112 ± 21	94 ± 17	1.8	0.181
*I*_max__S (mm)	61 ± 5	64 ± 4	58 ± 4	0.8	0.439
AUC					
AUC_R (ppbV.s)	6769 ± 1399	5857 ± 1145	4697 ± 956	1.2	0.333
AUC_S (mm.s)	8525 ± 780	8734 ± 834	7873 ± 775	0.6	0.553
*T*_max_					
*T*_max__R (s)	85 ± 6	90 ± 7	83 ± 5	0.4	0.421
*T*_max__S (s)	20 ± 3	22 ± 4	23 ± 4	0.1	0.884

(B) Beef	*BEEF60*	*BEEF70*	*BEEF90*	*F*	*p*
*I*_max_					
*I*_max__R (ppbV)	66 ± 13	100 ± 22	68 ± 11	2.0	0.138
*I*_max__S (mm)	47 ± 6	48 ± 6	43 ± 5	0.5	0.612
AUC					
AUC_R (ppbV.s)	3087 ± 670	4729 ± 1149	2980 ± 471	1.1	0.333
AUC_S (mm.s)	5420 ± 814	6615 ± 914	5315 ± 617	1.0	0.360
*T*_max_					
*T*_max__R (s)	83 ± 5	85 ± 5	82 ± 4	0.2	0.784
*T*_max__S (s)	23 ± 5	20 ± 4	18 ± 4	0.6	0.560

a The parameters *I*_max_, AUC, C_end_, and *T*_max_ correspond to the maximum concentration of released menthone (R) or maximum sensory peppermint intensity (S), the total amount of released menthone (R) or overall sensory peppermint intensity (S), and the time to reach the maximum released concentration of menthone (R) or the time to reach maximum sensory intensity of peppermint (S), respectively. *F* and *p* values are derived from linear mixed models with samples as fixed factors and participants, replicates, and order as random effects.

The *in vivo* menthone release curves for all PBMA (Figure 2A) and beef patties (Figure 2C) showed a steep increase in menthone release from the start of the mastication process until the first swallowing moment (dotted line). After the first swallowing moment, an additional increase in menthone release is observed, caused by the swallowing breath. Between the first and second swallowing moments (dotted and dashed lines), the menthone release decreased slightly for PBMA and beef patties. The swallowing breath was less pronounced after the second swallow (except for BEEF70), and the release of menthone decreased further until the end of the measurement. Overall, the release was lower for less juicy patties, although this trend was not completely consistent.

In agreement with the *in vivo* menthone release curves, the time–intensity curves for all PBMA (Figure 2B) and beef (Figure 2D) patties showed a steep increase in peppermint intensity from the start of consumption until the first swallowing moment. After the first swallowing moment, a slight increase in perceived intensity was observed, mostly for PBMA patties and especially for PBMA60, which corresponds to the swallowing breath. After the second swallowing moment, perceived peppermint concentration decreased, and this decrease is in line with the observed decrease in the release curves. The lingering effect of menthone was observed in all PBMA and beef patties, indicating its independence from the juiciness of the patties. The lingering effect is, therefore, more related to the properties of the aroma compound itself. Davidson et al. demonstrated a cross-modal interaction in which peppermint perception decreased with decreasing sucrose concentration while the in-nose release of menthone was constant during the mastication of chewing gum.^48^ No such interaction was found in our patties since the differences in juiciness did not affect menthone release across PBMA and beef patties. The differences in the menthone release patterns between the two studies can be explained by the differences in stimuli and the mastication protocols. Davidson et al. investigated chewing gum, which was chewed for 5 min without swallowing, so that menthone was continuously released during the mastication process, while mint perception declined over the course of 5 min. The decline in perception might be the result of adaptation effects and cross-modal interactions.^48^ The PBMA and beef patties were chewed for only 30 and 35 s, respectively, and then swallowed. This explains why the *in vivo* release of menthone tended to decline after 30 and 35 s for PBMA and beef patties, respectively, and why the perception of peppermint tended to decline at a faster rate after 30 and 35 s for PBMA and beef patties, respectively.

Although the curves in Figure 2 suggest that differences are obtained between samples, Table 5 shows that none of the parameters extracted from the *in vivo* menthone release and peppermint intensity profiles differed significantly across PBMA or beef patties varying in the core temperature. This demonstrates that the perceived variations in juiciness of PBMA and beef patties did not cause significant differences in menthone release and peppermint aroma perception, which confirms our hypothesis that an increase in juiciness increases *in vivo* aroma release and perception through variations in the degree of serum release. The descriptive sensory profiles obtained by the RATA profiling demonstrated that with decreasing core temperature (increasing juiciness), saltiness, umami, and meat flavor intensity of beef patties increased, whereas beany flavor intensity decreased (Table 3). For PBMA patties, with decreasing core temperature (increasing juiciness), off-flavor intensity decreased, whereas umami, saltiness, meat, and beany flavor intensity were not influenced by variations in juiciness (Table 2).

Our results contradict the results found in the literature. Previous studies demonstrated that increased marbling led to increased juiciness and, consequently, to differences in in-mouth volatile concentrations in grilled beef.^6,26^ However, the marbling provides a large influence on the composition and structure, which may explain why this study showed such a correlation. In addition, the difference may also come from the fact that the marbling releases more fat, which has an influence on the type of aroma compounds that will be released. The amount of released compounds will depend on the chemical nature of the aroma compound. For example, Frank et al. found that an increase in juiciness enhanced the release of hydrophilic compounds such as 2,3-butanedione and 2-butanone (Log *P* = −1.34 and Log *P* = 0.29), while hexanal (Log *P* = 1.78), a hydrophobic compound, did not show an increase in release.^6^ In the mentioned study, most of the released serum was probably water, while limited fat was present. In our study, the PBMA and beef patties were spiked with menthone, a hydrophobic compound (Log *P* = 2.7), selected for its detectability, absence in the headspace of the patties, and distinct peppermint aroma during consumption. The release of such compounds would, therefore, be enhanced when the serum contains fat. It has previously been shown that the majority of the serum released from similar PBMA and beef patties is fat.^23^ We therefore expected that menthone could have been easily released with the fat phase of the serum, which is squeezed out upon mastication. However, the amount of serum that was released from these patties during mastication was limited. At a core temperature of 90 °C, the serum release was only 3% w/w, which increased to 13% w/w at a core temperature of 60 °C.^23^ This corresponds to 0.36 and 1.56 g of serum. The vast majority of the serum, including both fat and water, therefore remained trapped inside the solid patty matrix (bolus), which limited the release of the compounds from the patties during consumption. This might explain why, in our study, variations in juiciness were too small to cause significant differences in menthone release and peppermint aroma perception.

Another reason for the lack of differences in menthone release and peppermint perception in the patties could be due to the interindividual differences between participants. Interindividual differences influence the *in vivo* release and perception of aroma.^29,49^ The current study took several measures to minimize interindividual differences between participants. However, the individual curves of the different participants indicated substantial variation between participants. This might have been prevented by taking even more severe measures to reduce interindividual differences, such as selecting participants from a narrower age range, applying stricter screening criteria, screening more physical parameters that affect oral breakdown, selecting participants with similar saliva composition, and including only specific ethnicities.

Future studies to clarify the role of juiciness and serum release on aroma release and perception could opt to select different ingredients and aroma compounds to prepare the PBMA patties to enlarge the differences between patties. For example, commercial PBMAs incorporate various types of fat, but their direct impact on juiciness, aroma release, and perception remains unclear. Moreover, fat influences the structure of the PBMA matrix and the composition of released serum. The composition of the serum could influence juiciness, aroma release, and perception of PBMAs, so variations in composition might affect aroma release and perception. Another option is to study the interaction between juiciness, aroma release, and perception by using aroma compounds differing in volatility and hydrophobicity. The current study included only one aroma compound (menthone) that was incongruent with the flavor of the food matrix. Future studies could opt to include aroma compounds of different chemical classes, since these compounds could be released differently from the PBMA matrix and have a different effect on aroma perception. Moreover, it is of interest to study the release and perception of aroma compounds that closely resemble meat flavor because then the aroma that is monitored would be congruent with the flavor of the patties. Such variations hopefully clarify relationships between different characteristics of complex food products such as beef and meat analogue patties.

To conclude, juiciness of commercial PBMA and minced beef patties was altered by varying the core cooking temperature during sous vide cooking. Although differences in juiciness were perceived, juiciness did not influence the *in vivo* release of menthone and the perception of peppermint in PBMA and beef patties. The limited amount of serum released from the patty matrix during mastication and the entrapment of fat in the bolus might have mitigated the hypothesized effect of juiciness on aroma release and perception. Interindividual differences between participants probably had a greater effect on aroma release and perception than differences between patties.

## Abbreviations

PBMA: plant-based meat analogue
TVP: textured vegetable protein
TI: time-intensity
PTR-MS: proton transfer reaction–mass spectrometry
RATA: Rate-All-That-Apply
ppbV: parts per billion by volume
AUC: area under the curve
*I*_max_: maximum intensity
*T*_max_: time of maximum intensity
SD: standard deviation
SE: standard error
LMM: linear mixed model
Log *P*: partition coefficient
